# Novel Mutations in *TACSTD2* Gene in Families with Gelatinous Drop-like Corneal Dystrophy (GDLD)

**DOI:** 10.22088/BUMS.6.4.204

**Published:** 2017-12-11

**Authors:** Elham Alehabib, Javad Jamshidi, Hamid Ghaedi, Babak Emamalizadeh, Monavvar Andarva, Narsis Daftarian, Mozhgan Rezaei Kanavi, Peyman Mohammadi Torbati, Goldis Espandar, Somayeh Alinaghi, Amir Hossein Johari, Mansoor Saghally, Fatemeh Mohajerani, Hossein Darvish

**Affiliations:** 1 *Department of Medical Genetics, School of Medicine, Shahid Beheshti University of Medical Sciences, Tehran, Iran.*; 2 *Noncommunicable Diseases Research Center, Fasa University of Medical Sciences, Fasa, Iran.*; 3 *Department of Medical Genetics, Faculty of Medicine, Tabriz University of Medical Sciences, Tabriz, Iran.*; 4 *Ocular Tissue Engineering Research Center, Shahid Beheshti University of Medical Sciences, Tehran, Iran.*; 5 *Department of Pathology, Labbafi-Nezhad Hospital, Shahid Beheshti University of Medical Sciences, Tehran, Iran.*

**Keywords:** Gelatinous drop-like, corneal dystrophy, GDLD; TACSTD, Iranian

## Abstract

In the current study, we conducted a mutation screening of tumor-associated calcium signal transducer 2 (*TACSTD2*) gene in six consanguineous Iranian families with gelatinous drop-like corneal dystrophy (GDLD), in order to find the causative mutations. Detailed eye examination was performed by ophthalmologist to confirm GDLD in patients. To detect the possible mutations, direct Sanger sequencing was performed for the only exon of *TACSTD2* gene, and its boundary regions in all patients. In the patients with GDLD, the corneal surface showed lesions with different shapes from mild to severe forms depending on the progress of the disease. The patients showed grayish corneal deposits as a typical mulberry form, corneal dystrophy along with corneal lipid deposition, and vascularization. Targeted Sanger sequencing in *TACSTD2* gene revealed the causative mutations in this gene in all studied families. Our study expanded the mutational spectrum of *TACSTD2* which along with the related symptoms could help with the diagnosis, and management of the disease.

Gelatinous drop-like corneal dystrophy (GDLD) (OMIM#204870) is a type of eye disorder in which the patients lose their vision because of amyloid deposition on the cornea. Based on the international committee for classification of corneal dystrophies (IC3D) classification, GDLD is a member of sub epithelial dystrophies (1). This rare disorder is inherited as an autosomal recessive trait. GDLD appears in the first or second decade of life, and usually leads to blindness as its natural course (2). It is well-known that the causative gene for GDLD is tumor-associated calcium signal transducer 2 (*TACSTD2*), which was identified by Tdujikawa et al. in 1998 (3). *TACSTD 2 *has one exon with 972 nucleotides, and encodes for a protein with 323 amino acids (3). TACSTD 2 acts as a calcium signal transducer, and has receptor function activity (4). In patients with GDLD, superficial cells in cornea do not express claudin 1, tight junction protein 1, and occludin, in addition to stability change in cell-to-cell junctions. These findings suggest that any pathogenic alterations in *TACSTD2* gene, due to loss of function, changes the permeability in epithelial cells that lead to clinical symptoms in cornea (5). Decreased epithelial barrier function, eases lactoferrin -an iron- binding glycoprotein present in exocrine fluids- deposition on the cornea, which eventually leads to amyloid deposition (6-7). It has been reported that the phenotypic variability in GDLD could be due to age-related progression over time (8). However, a recent study in a consanguineous Colombian family with 5 affected patients suggested that environmental or other genetic factors may also affect the manifestation of clinical symptoms (8). The other aspect of *TACSTD2* gene, is its over expression relation with tumor cells. The role of *TACSTD2* gene in progression and aggressiveness of colorectal cancer has been confirmed in different studies (9-12), but coincidence of colorectal cancer in patients with GDLD has been less considered. Prevalence of GDLD has not been determined in Iran, and previous studies have revealed some pathogenic mutations in Iranian population. In the current study, we investigated six consanguineous Iranian families with GDLD to detect the causative mutations and explain the symptoms related to each mutation and family.

## Materials and methods

***Subjects***

This is a descriptive study conducted in six consanguineous Iranian families with GDLD referred from Mazandaran, Khuzestan, Tehran and Isfahan to Labbafinejad Hospital (Tehran, Iran). Clinical investigations were performed according to the Declaration of Helsinki, and all the families signed the informed consent for participating in this study. The Ethical Committee of Shahid Beheshti University of Medical Sciences approved this study.

**Fig. 1:**
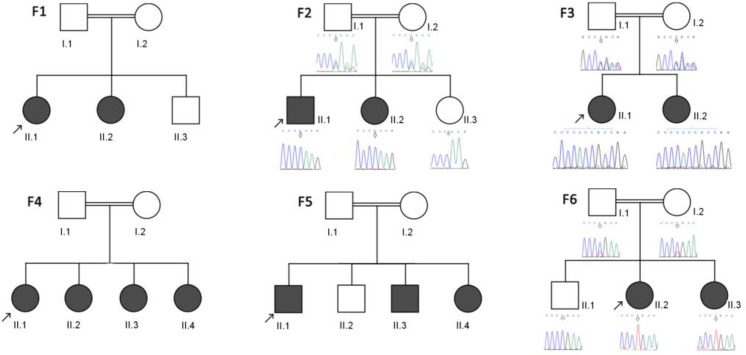
Pedigrees of the six studied families with GDLD. Partial sequences of the mutation location is shown in the cases of novel mutations (P.Lys84Glnfs*11 and p.Gly165Profs*15 related to F.2 and F.3, and p.Pro266Leu, in F.6

Six consanguineous Iranian families with at least two patients with GDLD were involved in our study. The pedigrees of these families are shown in Figure 1. The diagnosis of GDLD was performed by ophthalmologist, based on clinical manifestations, ocular examination, and histopath-ology evaluation of cornea tissue.

***Ocular examinations***

Uncorrected visual acuity was performed using the standard 20 feet Snellen acuity chart. Best corrected visual acuity tests, and intraocular pressure were not possible to undertake because of severe photophobia and corneal opacity. Complete slit-lamp bio microscopy examinations including the conjunctiva, cornea, anterior chamber clarity, angle and iris were done as much as possible by the corneal opacity evaluation.

***Histopathology assessment***

Patients’ corneas, fixed in 10% formalin were bisected, processed, and embedded in paraffin blocks. Thin tissue sections were stained with hematoxylin and eosin, periodic acid-Schiff (PAS), Masson’s Trichrome, and Congo-red staining methods. All stained slides were examined under light microscopy (BX41, Olympus, Japan), and photographed with a digital camera (DP12 Microscope Camera, Olympus, Japan).

***Molecular analysis***

DNA was obtained from white blood cells in peripheral blood using a standard salting-out method (13). Three pairs of primers were designed for the only exon of *TACSTD2* using primer 3 program. The sequence of the three primer pairs are as follows: first pair (Forward: 5′-TGCAGACCAT-CCCAGACG-3′, Reverse: 5′-TAGAGGCCATC-GTTGTCCAC-3′) second pair (Forward: 5′-CAAGTGTCTGCTGCTCAAGG-3′, Reverse: 5′-CTCGATCTGGATGGTGGGC-3′) and third pair (Forward: 5′-GCTGCACCCCAAGTTCGT-3′, Re-verse: 5′- GACTCACTTGGGTCTGGGAC- 3′). Polymerase chain reaction (PCR) was performed for all patients’ samples, and products were sequenced by directed Sanger sequencing with Applied Biosystems BigDye terminator v3.1 sequencing chemistry on an ABI3130 genetic analyzer (Applied Biosystems, Foster City, CA, USA). Then, the sequence data were compared to reference sequence of the gene, available at the National Center for Biotechnology Information (NCBI), and analyzed using Sequencer 5.0 software (Gene Codes Corporation, Ann Arbor, MI, USA). Familial segregation of the mutations, in each family was also performed by targeted Sanger sequencing of the gene in other family members. The detected mutations were also confirmed using sequencing with the reverse primer.

**Fig. 2:**
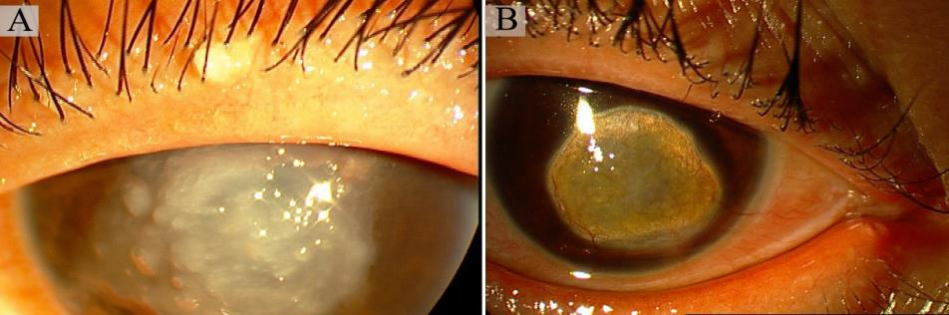
Slit lamp photographs of the corneas. A: grayish corneal deposits are visible as typical mulberry form gelatinous corneal dystrophy. Note that the patient was not able to open her eye completely because of blepharospasm secondary to photophobia. (F.4, P2); B: mulberry form corneal dystrophy along with corneal lipid deposition and vascularization are visible as a result of repeated lamellar keratectomies (F.4, P3

***Bioinformatics analysis***

To assess the potential functional impact of the Glu227Lys and Pro266Leu, we utilized four widely used algorithms to perform *in silico* analysis: SIFT (sorting intolerant from tolerant), PolyPhen-2 (polymorphism phenotyping-2), Align GVGD (align Grantham variation Grantham deviation), and I- Mutant v.2. In general, these four tools all have distinct algorithms with variable, however, high prediction accuracy.

**Fig. 3:**
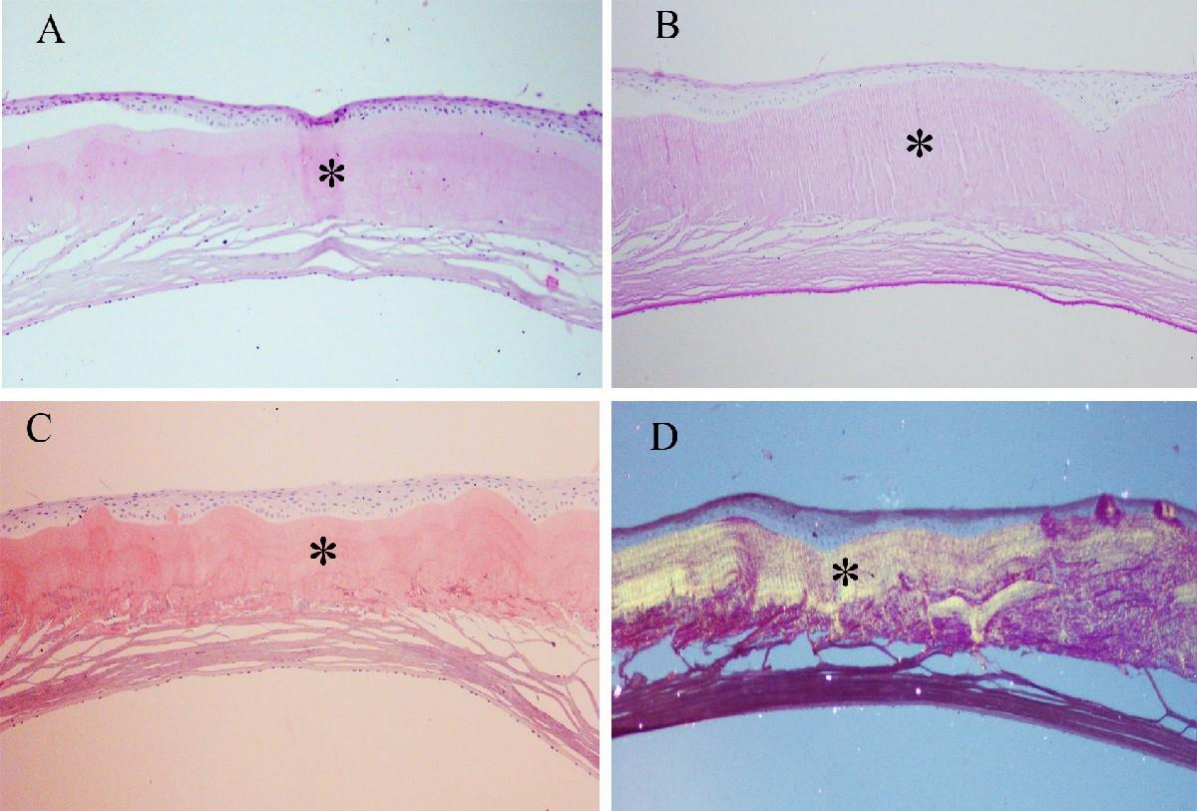
Photomicrographs of full thickness cornea in the patient with GDLD. Note the presence of prominent amorphous and eosinophilic material (asterisk) in the sub epithelial area on hematoxylin and eosin (A), with mild PAS-reactivity (B, asterisk), and positive staining for Congo red (C, asterisk). The sub epithelial deposits reveal a red apple green birefringence (D, asterisk) under polarized light (all images magnification × 100

**Fig. 4:**
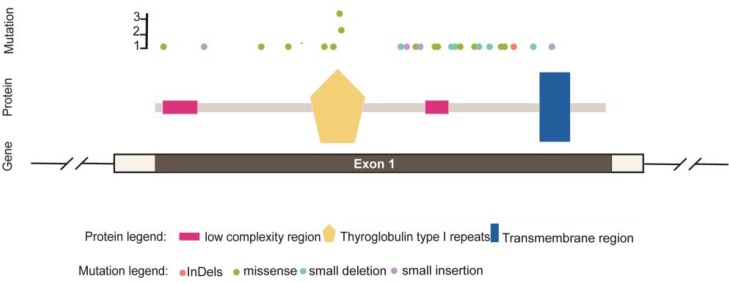
The schematic representation of mutations spectrum in *TACSTD2*

## Results

***Phenotype of patients***

Six consanguineous Iranian families with GDLD were studied. The clinical characteristics of patients in each family are presented in table 1. Slit lamp photographs of the corneas (related to II.2 and II.3 in family 4) are presented in Figure 2. Slit lamp photographs in the other affected individuals were not performed because of no significant clinical feature or surgical operation.

Histopathological examinations disclosed full cornea thickness with a prominent band-shaped eosinophilic amorphous material underlying an irregular corneal epithelium. The deposits were slightly PAS-reactive, and stained positively on Congo red which revealed a red apple green birefringence-the sign of the presence of amyloid fibrils- under polarized light (Figure 3). The microscopic features were diagnostic of a GDLD.

***TACSTD2*** ***mutation analysis***

GDLD-causing mutations were detected in all the patients within *TACSTD2* gene (Table 1). Five different mutations were found including: p.Cys 66*, p.Glu 227Lys, p.Lys 84Glnfs*11, p.Gly 165 Profs*15, and p.Pro 266 Leu. The nucleotide alteration which caused the amino acid substitution, Pro266Leu, was also assessed in 100 unrelated control individuals by targeted Sanger sequencing, and was not observed in them. 

Pathogenicity predictions for novel missense mutations identified in our study (p.Glu227Lys, and p.Pro266Leu) are presented in table 2.

## Discussion

In the current study, we investigated six Iranian families with GDLD. We screened *TACDTD2 *gene to detect the possible mutations related to this condition in our patients. The pathogenic mutations were found in all six studied families in *TACDTD2 *gene. Two of these mutations (p.Cys66*, p.Glu227Lys) were previously reported (14) and three of them (p.Lys84Glnfs*11, p.Gly165Profs*15 and p. Pro266Leu) were novel based on Exome Aggregation Consortium (ExAC Browser), NHLBI Exome Variant Server, dbSNP and 1000 genomes databases.

**Table 1:** Clinical Features of GDLD patients.

**Family**	**Current age**	**Age of onset**	**Sex**	**Mutation**	**BCVA (Snellen)**	**Phenotypic expression (Type of Amyloidosis)**	**Vascularization**	**Site of Amyloidosis**	**Unilateral /bilateral affected**	**Relative Severity**	**Progression (Recurrence** ^*^ **)**	**Treatment strategies**
F.1 P1	20 y	12 y	F	c.198C>A (p.Cys66*)	C.F.	mulberry	No	Total cornea	Bilateral	severe	Progressive & recurrency	Lamellar Keratectomy
F.1 P2	18 y	6 y	F		C.F.	mulberry	No	Total cornea	Bilateral	severe	Progressive & recurrency	Lamellar Keratectomy
F.2 P1	28 y	15 y	M	c.249_250insC (p.Lys84Glnfs*11)	C.F.	mulberry	No	Total cornea	Bilateral	severe	Progressive & recurrency	Penetrating Keratoplasty
F.2 P2	25 y	13 y	F		C.F.	mulberry	No	Total cornea	Bilateral	severe	Progressive & recurrency	Penetrating Keratoplasty
F.3 P1	17 y	16 y	F	c.492_493insCCACCGCC(p.Gly165Profs*15)	C.F.	mulberry	No	Central	Mon lateral	severe	Progressive & recurrency	SK:OU, LK:OU
F.3 P2	12 y	6 y	F		C.F.	mulberry	No	Central	Bilateral	severe	Progressive & recurrency	SK:OU, LK:OU
F.4 P1	47 y	at birth	F	c.679G>A (p. Glu227Lys)	Hand motion (both eyes)	Severe form of typical mulberry type corneal dystrophy	Corneal vascularization and severe lipid deposition	Central corneal	Bilateral	Severe	Progressive	Multiple times corneal lamellar keratectomy (both eyes)
F.4 P2	45 y	2 y	F		Hand motion (both eyes)	Severe form of typical mulberry type corneal dystrophy	No	Central corneal	Bilateral	Severe	Progressive	Conservative
F.4 P3	35 y	2 y	F		Counting finger at 20 cm (RE) Hand motion (LE)	Not evaluable because of previous surgeries	Corneal vascularization and mild corneal lipid deposition	Central	bilateral	Severe	Recurrency in both eyes after surgeries	Lamellar keratectomy(RE) Penetrating keratoplasty (LE)
F.4 P4	31 y	12 y	F		20/100 (RE) Hand-motion (LE)	Typical mulberry type corneal	No	Central	Bilateral	Severe	Progressive and recurrency	Lamellar keratectomy (both eyes)
F.5 P1	55 y	12 y	M	c.679G>A (p. Glu227Lys)	RE: LP, LE: CF	Severe, mulberry	Corneal vascularization and mild corneal lipid deposition	Central	bilateral	severe	Progressive & recurrency	Multiple times corneal lamellar keratectomy (both eyes)
F.5 P2	45 y	3 y	M		RE: LP, LE: CF	Severe, mulberry	Corneal vascularization and mild corneal lipid deposition	Central	bilateral	severe	Progressive & recurrency	Multiple times corneal lamellar keratectomy (both eyes)
F.5 P3	38 y	6 month	F		RE :LP, LE: CF	Severe, mulberry	Corneal vascularization and mild corneal lipid deposition	Central	bilateral	severe	Progressive & recurrency	Multiple times corneal lamellar keratectomy (both eyes)
F.6 P1	50 y	7 y	F	c.797C>T (p. Pro266Leu)	C.F.	Typical mulberry type corneal	No	Central	bilateral	severe	Progressive & recurrency	Penetrating Keratoplasty
F.6 P2	45 y	7 y	F		C.F.	Typical mulberry type corneal	No	Central	bilateral	severe	Progressive & recurrency	Penetrating Keratoplasty

BCVA: Best Corrected Visual Acuity; CF: Counting Fingers; F: Female; LE: Left Eye; LP: Light Perception; M: Male; RE: Right Eye; Y: year**.**

**Table 2:** Pathogenicity predication for novel missense mutations identified

Mutation	SIFT score (prediction)	Polyphen score (prediction)	Align GVGD	I-mutant stability score (prediction)
Glu227Lys	0 (damaging)	1.00 (probably damaging)	damaging (Class 55)*	6 (decreased)
Pro266Leu	0.01 (damaging)	1.00 (probably damaging)	damaging (Class 65)*	5 (decreased)

*TACSTD2 *codes for a protein with a single-transmembrane domain (AA 275–297), an epidermal growth factor-like repeat, a thyroglobulin domain, and a phosphatidylinositol 4, 5-bis phosphate-binding domain. This monomeric cell surface glycoprotein is present in organs such as cornea, placenta, trophoblasts, pancreas, kidney, pancreas, prostate, lung, and colon. Expression of this gene has been also reported in some carcinomas (14). *TACSTD2* is a CpG rich gene, and has 22 SNPs, which may affect the function, and expression of this gene. Due to the presence of CpG islands in the majority of *TACSTD2* regions, it seems that expression of this gene is impressed by epigenetic regulation (7).

p.Cys66* mutation, which was found in family 1, causes a truncated protein after about one fifth of the mRNA translation. In the previous report, patient with this mutation had not shown the initial symptoms in early life, and the clinical signs started at the second decade of life (15). In our family with the same mutation, the age of onset was earlier, and it was also different between affected individuals in the family.

p.Lys84Glnfs*11 and p.Gly165Profs*15, two of the novel mutations, are the consequence of nucleotide insertion, which leads to a frame shift, early stop codon, and truncated protein. p.Gly165Profs*15, p.Glu227Lys, and p. Pro266Leu occurred between the thyroglobulin-like (TY) and transmembrane (TM) domains of the protein. According to the schematic representation of mutation spectrum in *TACSTD2* gene (Figure 4), which is prepared in accordance with reported mutations up to now, about 50 percent of all deleterious mutations in *TACSTD2* gene occur between the TY and TM domains. The exact function of this region is not known (15).

The mutation in family 3 was an eight-nucleotide insertion (c.492_493insCCACCGCC (p.Gly165Profs*15)) that leads to a frame shift. The two patients in this family manifested the disease differently; in addition to different age of onset, in one of them both eyes were involved, and in another patient only one eye was involved, although she may develop the disease to the other eye later in her life.

p.Glu227Lys which was detected in two families in our study (F4 and F5), has been reported before, and was introduced as a founder mutations in Iranian population (15). Glutamic acid in this site is conserved in *TACSTD2* gene, and located between TY-TM domains. We saw variable phenotypic expression, and different age of onset in patients with this mutation in the same family, and also between the patients of the two families (F4 and F5). This mutation is one of the nucleotide alterations that are related with severe form of GDLD, because the patients with this mutation experience the amyloid deposition in early life with severe form of GDLD. A study also has reported paracentral amyloid deposition on the cornea in some patients with this mutation, which may indicate the involvement of other factors in determining the clinical manifestation of the disease (15). An interesting finding is that one of our patients with this mutation (F5P2) had history of intestinal problems with the diagnosis of adenocarcinoma. He had colonoscopy for removing the polyps. Association of adenocarcinoma with GDLD was also reported previously for a different mutation (p.Tyr184Cys) (8).

p. Pro266Leu, as a novel mutation, was discovered in family 6, and the pathogenicity of this amino acid substitution was confirmed with bioinformatics tools. Moreover, this mutation was not seen in 100 normal individuals examined for this alteration, which support its pathogenicity.

Deleterious homozygous mutations, such as the mutations in our families, usually cause loss of function in *TACSTD2* gene. To date about 33 mutations, in *TACSTD2* gene have been reported from different geographical regions including Japan, Iran, India, Tunisia, China, Estonia, Turkey, Vietnam, Colombia, Latin America, Thailand, and Europe (6, 8, 14-22). The evidence from Japan suggests that the prevalence of GDLD is higher in this country, about 1 in 31,000 (23).

Some patients with amyloid deposition on the cornea have been reported previously that did not show pathogenic nucleoid alteration in *TACSTD2* gene. In these cases, two other candidate genes transforming growth factor beta induced  (*TGFBI*) and gelsolin (*GSN*) were assessed. Evidence showed that pathogenic nucleotide alteration in these two genes also lead to amyloid deposition, although their inheritance, and clinical signs are different from GDLD (14).

In conclusion, in the current study, we introduced three novel mutations in *TACSTD2* gene which cause GDLD. Our study expanded the mutational spectrum of *TACSTD2* gene, which along with the related symptoms could help with the diagnosis and management of the disease. Functional studies are needed to clarify the molecular mechanisms which lead to GDLD due to mutations in *TACSTD2* gene.
